# Obstructive sleep apnea and cortical thickness in females and males

**DOI:** 10.1371/journal.pone.0193854

**Published:** 2018-03-06

**Authors:** Paul M. Macey, Natasha Haris, Rajesh Kumar, M. Albert Thomas, Mary A. Woo, Ronald M. Harper

**Affiliations:** 1 UCLA School of Nursing, University of California at Los Angeles, Los Angeles, CA, United States of America; 2 Brain Research Institute, David Geffen School of Medicine at UCLA, University of California at Los Angeles, Los Angeles, CA, United States of America; 3 Department of Anesthesiology, David Geffen School of Medicine at UCLA, University of California at Los Angeles, Los Angeles, CA, United States of America; 4 Department of Radiological Sciences, David Geffen School of Medicine at UCLA, University of California at Los Angeles, Los Angeles, CA, United States of America; 5 Department Neurobiology, David Geffen School of Medicine at UCLA, University of California at Los Angeles, Los Angeles, CA, United States of America; University of Rome Tor Vergata, ITALY

## Abstract

**Introduction:**

Obstructive sleep apnea (OSA) affects approximately 10% of adults, and alters brain gray and white matter. Psychological and physiological symptoms of the disorder are sex-specific, perhaps related to greater injury occurs in female than male patients in white matter. Our objective was to identify influences of OSA separated by sex on cortical gray matter.

**Methods:**

We assessed cortical thickness in 48 mild-severe OSA patients (mean age±std[range] = 46.5±9.0[30.8–62.7] years; apnea-hypopnea index = 32.6±21.1[6–102] events/hour; 12 female, 36 male; OSA severity: 5 mild, 18 moderate, 25 severe) and 62 controls (mean age = 47.7±8.9[30.9–65.8] years; 22 female, 40 male). All OSA patients were recently-diagnosed via polysomnography, and control subjects screened and a subset assessed with sleep studies. We used high-resolution magnetic resonance imaging to identify OSA-related cortical thinning, based on a model with condition and sex as independent variables. OSA and OSA-by-sex interaction effects were assessed (*P*<0.05, corrected for multiple comparisons).

**Results:**

Multiple regions of reduced cortical thickness appeared bilaterally in the superior frontal lobe in female OSA vs. all other groups. Significant thinning within the pre- and post-central gyri and the superior temporal gyrus, extending into the insula, appeared between the general OSA populations vs. control subjects. No areas showed increased thickness in OSA vs. controls or positive female OSA interaction effects.

**Conclusions:**

Reduced cortical thickness likely represents tissue atrophy from long term injury, including death of neurons and supporting glia from repeated intermittent hypoxic exposure in OSA, although disease comordities may also contribute to thinning. Lack of polysomnography in all control subjects means results may be confounded by undiagnosed OSA. The greater cortical injury in cognitive areas of female OSA patients may underlie enhanced symptoms in that group. The thinning associated with OSA in male and females OSA patients may contribute to autonomic dysregulation and impaired upper airway sensori-motor function.

## Introduction

The repeated collapse of the upper airway in Obstructive Sleep Apnea (OSA) leads to neural changes from the resulting intermittent hypoxia and cardiovascular sequelae, which then result in psychological and physiological deficits, including increased sympathetic tone [1–3]. Symptoms and brain changes in OSA show sex differences, with female OSA patients exhibiting greater white matter injury than males, and more severe impairments in some neurological functions [4, 5]. Whether cortical gray matter is compromised similarly to white matter is unknown. The OSA-related injury in white matter will affect communication between cortical and subcortical structures, and almost certainly affect the cortical neurons from which those axons emerge.

Males have a two-fold higher incidence of OSA than females [6]. However, OSA characteristics differ between males and females. Prior studies suggest possible gender differences: Females with the disorder have a reported higher prevalence of chronic pulmonary disease, heart failure, tissue fluid retention, and hypothyroidism, while male OSA patients showed higher levels of cardiovascular disease and arrhythmia than females [7]. Additionally, females with OSA have a higher probability of experiencing neuropsychological disorders than males, including depression, insomnia and anxiety [8, 9]. Women with OSA, on average, have lower severity [10], but display more marked symptoms for equivalent condition severity (as measured by the apnea/hypopnea index, or AHI) [5, 10]. Since many OSA symptoms derive from central nervous system alterations, disparities in the expression of such symptoms may develop from sex differences in brain function and injury. The specific enhancement of axonal injury in OSA females appears in structures that serve a range of emotional, breathing and sleep integrity-mediating structures [4]. Since axonal injury is usually accompanied by damage to its cell body [11], such female-specific injury likely extends into cortical regions.

Injury specific to the cortex can be assessed by evaluating cortical thickness calculated from high resolution anatomical brain magnetic resonance imaging (MRI) scans; a set of software tools, FreeSurfer, has been developed to evaluate such brain structural properties [12]. The suite allows calculation and statistical analysis of group variations in cortical thickness at different locations. Reductions in cortical thickness reflect atrophy [13], which in OSA could arise from accelerated degeneration, including loss of neurons and glia [14].

The objective was to assess cortical thickness in separately female and male OSA and control subjects, based on high resolution MRI scans processed with FreeSurfer. We hypothesized that females with OSA would have decreased thicknesses in cortical areas, relative to male OSA and control participants, especially those areas associated with depression and anxiety, symptoms preferentially more common in females with OSA [8, 9]. Since OSA is accompanied by a range of behavioral and physiological deficits, as well as structural brain changes in mixed patient populations, differences in cortical thickness between OSA and control subjects were also expected.

## Methods

### Patients

We studied a total of 110 subjects, which included 12 female (mean age±SEM [range] = 51.3±2.3 [37.0–62.2] years; mean apnea-hypopnea index [AHI] ±std = 26.7±7.2 [5–89.3] events/hour) and 36 male (mean age = 45.0±1.5 [30.8–62.7] years; mean AHI = 34.6±3.3 [10–100.7] events/hour) recently-diagnosed OSA patients (AHI ≥ 5), and 22 female (mean age = 50.7±1.7 [40.2–65.8] years) and 40 male (mean age = 46.0±1.4 [30.9–64.5] years) control subjects. OSA patients underwent a standard overnight in-lab polysomnographic study within three months of enrollment. The scoring was based on 1999 American Academy of Sleep Medicine criteria, including a definition of apnea/hypopnea of lasting 10 seconds or longer, and involving a desaturation of >3% SaO_2_ in the case of a hypopnea, although most apneas also show desaturations [15]. OSA subjects were not being treated for their condition, nor were they taking any cardiovascular or psychotropic medications. Control subjects were recruited from the neighborhood of UCLA via fliers, and were in good health with no reported sleep problems. All control participants were screened for indications of OSA by questionnaires and interviews, and based answers to those questions (high sleepiness, snoring), six were further assessed with an overnight polysomnographic study. Two of those were confirmed as having normal sleep, two as having OSA, and two has having mild sleep-disordered breathing not meeting criteria for OSA. All participants were free of metallic implants, and exclusion criteria included history of mental illness, history of tobacco use, other major disease (cancer, major cardiovascular events, stroke, neurological disorders, chronic obstructive pulmonary disease), or head injury. No participants were diagnosed with hypertension, although the condition was likely present in at least some OSA patients. At the time of recruitment, no particiapnts were known to have diabetes, but subsequently four OSA patients were found to have Type II diabetes. The study was approved by the UCLA Institutional Review Board, and participants provided written informed consent.

### Sleepiness and neuropsychological questionnaires

We administered the Epworth Sleepiness Scale (ESS) questionnaire to assess daytime sleepiness [16]. This test is scored between 0 and 24, with any score greater than or equal to 10 classified as outside the range of normal. We used the Pittsburgh Sleep Quality Index (PSQI), a 19-item questionnaire that assessed self-reported sleep quality and sleep times, with a score greater than or equal to 5 signifying clinically relevant disturbed sleep. The Beck Depression Inventory (BDI), a 21-item questionnaire, assessed depressive symptoms with scores greater than or equal to 10 indicating greater levels of depressive symptoms [17]. Lastly, the Beck Anxiety Index (BAI), a 21-item questionnaire, assessed levels of anxiety with scores greater than or equal to 8 indicating enhanced anxiety levels [18].

### MRI scanning

Brain scans were collected using a 3.0-Tesla MRI scanner (Magnetom Tim-Trio; Siemens, Erlangen, Germany) with a receive-only 8-channel phased-array head-coil and a whole-body transmitter coil. Foam pads were placed on either side of the head to reduce head motion related artifacts, and subjects lay supine during data collection. We collected two high-resolution T1-weighted image scans using a magnetization prepared rapid acquisition gradient-echo (MPRAGE) pulse sequence [repetition time (TR) = 2200 ms; echo time (TE) = 2.2 ms; inversion time = 900 ms; flip angle (FA) = 9°; matrix size = 256 × 256; field of view (FOV) = 230 × 230 mm; slice thickness = 1.0 mm; slices = 176].

### Cortical thickness processing

Data were preprocessed using SPM8. The two T1-weighted images were realigned and averaged, and rigid-body shifted into the common space.

We used FreeSurfer version 5.3.0 to process the averaged scans from each subject [19]. The initial skull stripping and boundary identification were performed, and skull strips of all subjects were manually assessed to ensure no brain areas were excluded or incorrectly included. Similarly, the pial and gray-white matter boundaries were visually assessed, and, if needed, edited to correct misidentified regions. To minimize bias, researchers not involved in the study performed the editing. Minor edits were required to adjust the automatically detected pial boundaries in most subjects, but the skull strip and white matter boundaries did not require adjustment.

### Statistical modeling

The FreeSurfer processing stream was followed to determine cortical thickness across the brain, excluding cerebellar areas, using a general linear model with condition (OSA/control) and sex as classification variables (10 mm smoothing). The linear model was implemented at each surface point. The model assessed cortical thinning as a function of condition, sex and their interaction, and was then interrogated to test specific hypotheses across the cortical surface. We corrected for multiple comparisons with Monte Carlo simulation with a threshold of *P* = 0.05. Results were displayed as colored “clusters,” that is, regions of adjoining surface points showing statistically significant differences, with color indicating statistical significance. We overlaid the areas of significant differences onto the pial cortical surface, and lateral views of the “inflated” surface (sulcal and gyral areas displayed as smooth adjacent regions without depth).

The FreeSurfer annotations were provided in table descriptions for consistency with the Desikan atlas provided in that software [20], although the terms do not always follow conventional anatomical labeling conventions. Hence, Brodmann areas (BAs) were also denoted.

## Results

### Demographics

Demographic data are listed in Table 1. Body mass indices were significantly higher in OSA females and OSA males, compared to their respective controls. There were no significant differences between the control and OSA groups, separated by sex, in age or years of education (age, *P* = 0.5; education, *P* = 0.2).

**Table 1:** Characteristics of OSA and control subjects, separate by sex, with group averages and standard deviations. Significance levels for ANOVA tests are shown.

		Female		OSA vs Control	Male		OSA vs. Control
Variable Type	Variables	Control	OSA	*P*	Control	OSA	*P*
		(Mean ± SEM)	(Mean ± SEM)	(F-test or Chi square)	(Mean ± SEM)	(Mean ± SEM)	(F-test or Chi-square)
Demographic	N	22	12	-	40	36	-
	Age (years)	50.7±1.7	51.3±2.3	0.84	46.0±1.4	45.0±1.5	0.62
	Education (years)	16.6±1.4	16.0±0.0	0.89	18.5±1.4	19.7±0.9	0.47
Biophysical	BMI (m^2^/kg)	23.9±1.1	32.9±1.7	<0.001	25.4±0.4	29.6±0.8	<0.001
	Handedness	16 right, 6 left	10 right, 2 left	0.49	35 right, 5 left	32 right, 4 left	0.85
Neuropsychological	BDIcog	1.95±0.5	7.00±2.2	0.0049	2.45±0.7	3.97±0.7	0.13
	BDIsom	2.41±0.7	6.00±1.6	0.020	1.78±0.3	4.64±0.6	<0.001
	BDI (Depression)	4.45±1.0	13.83±3.4	0.0025	4.20±0.9	8.61±1.2	0.004
	Beck Anxiety	4.5±1.2	19.8±4.4	0.0002	3.65±0.7	8.58±1.6	0.005
Sleep	Epworth SS	6.59±0.7	12.5±1.5	0.0002	5.08±0.6	9.89±0.8	<0.001
	PSQI	3.91±0.6	10.17±1.2	<0.001	3.80±0.4	9.31±0.7	<0.001
Polysomnography	AHI (events/hour)	-	26.7±7.2	-	-	34.6±3.3	-
			Range: 6–89			Range: 12–102	
	OSA severity distribution	-	4 mild, 3 moderate, 5 severe	-	-	1 mild, 15 moderate, 20 severe	-
	O_2_ saturation nadir (%)	-	86.3±1.8	-	-	78.2±1.6	-
	O_2_ saturation baseline (%)	-	94.7±0.5	-	-	94.9±0.3	-

### Cortical thinning: Effect of OSA independent of sex

**Fig 1:**
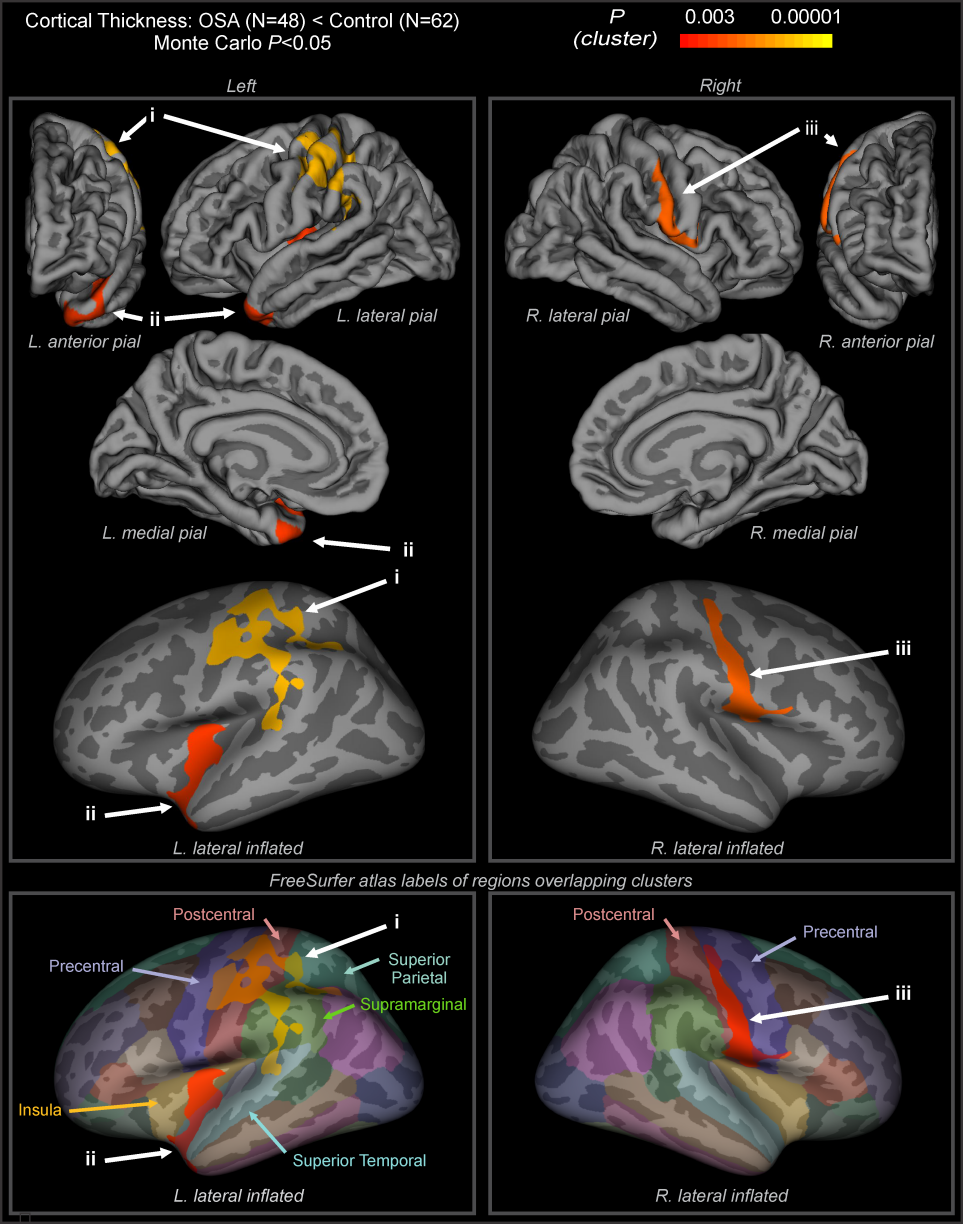
Cortical thinning in OSA. Regions showing cortical thinning in OSA compared to controls, accounting for sex (*p* < 0.05, Monte Carlo correction for multiple comparisons). Color scale shows the cluster-level *p* value. Hemisphere views include lateral, medial, frontal, and posterior. Pial and inflated views depicted, with light gray indicating gyri and dark gray sulci. (i) left hemisphere cluster extending into postcentral gyrus from lips and face to fingers sensory areas, precentral gyrus from neck to hand motor areas, supramarginal gyrus, Wernicke’s area, and superior temporal lobe; (ii) left hemisphere cluster extending into mid and posterior insula, temporal pole; (iii) right hemisphere cluster extending along the postcentral gyrus from upper airway to hand sensory areas, precentral gyrus in pharynx/tongue to lip motor areas. See Table 2.

Whole-brain group comparisons revealed cortical thinning associated with OSA accounting for sex as a covariate, compared to the control group in three defined clusters, after the Monte Carlo cluster correction was performed (*P* < 0.05). Affected areas included the left precentral gyrus (Talairach peak: -35.0, -12.0, 40.3), the left superior temporal gyrus and insular cortex (Talairach peak: -35.5, -12.8, 0.4), and the right postcentral gyrus (Talairach peak x, y, z mm: -56.3, -8.3, 11.2; Table 2 & Fig 1). Representations of areas of cortical thickness differences between control and OSA groups in the left and right hemispheres are shown in Fig 1 from multiple views overlaid onto the FreeSurfer template pial surface, and a lateral, inflated view. No regions showed increased cortical thickness in OSA patients.

**Table 2:** Areas of cortical thickness differences between OSA and control groups, as illustrated in Fig 1. Areas are labelled according to the Desikan [20] and Brodmann [70] atlases.

Anatomical Location of Cluster	Functional structures in cluster	Previous OSA/control findings overlapping this cluster	Figure	Cluster size (mm^2^)	Peak (Talairach coordinates in mm)			
					X	Y	Z	P*
• L. precentral gyrus^a^ • (BA 4) • L. postcentral gyrus (BA 1, 2, 3) • L. superior temporal gyrus (BA 41, 42) • L. superior parietal lobe (BA 5, 7) • L. supramarginal gyrus (BA 40) • L. inferior parietal lobe (BA 39)	• L. partial coverage of mid sensory gyrus (lips, face, hand) • L. mid motor gyrus (neck to wrist) • L. superior temporal gyrus: Wernicke’s area, speech perception/upper airway • L. supramarginal gyrus: language perception and processing • L. inferior parietal lobe: integration of visual and somatosensory information, maintaining attention [71], interpretation of emotions [72]	[46], [45], [43]	Fig 1i	3685	-35.0	-12.0	40.3	0.00010
• L. insula (BA 13, 14, 15, 16) • L. temporal pole (BA 38)	• L. insula posterior short gyrus/long gyrus (autonomic regulation, interoception & emotion) • L. temporal pole: semantic memory [73]	[73]	Fig 1ii	1485	-35.5	-12.8	0.4	0.00700
• R. postcentral gyrus^a^ (BA 1, 2, 3)* • R. precentral gyrus (BA 4)	• R. sensory gyrus in head/airway region (neck to tongue / swallowing) • R. inferior motor gyrus (tongue/upper airway)	[44]	Fig 1iii	1822	56.3	-8.3	11.2	0.00190

Abbreviations: L, left; R, right

a, FreeSurfer principal anatomical location of the peak (label of region most representative of area)

BA: Brodmann’s area

* cluster-wise P-value.

### Cortical thinning: Sex and OSA interaction

**Fig 2:**
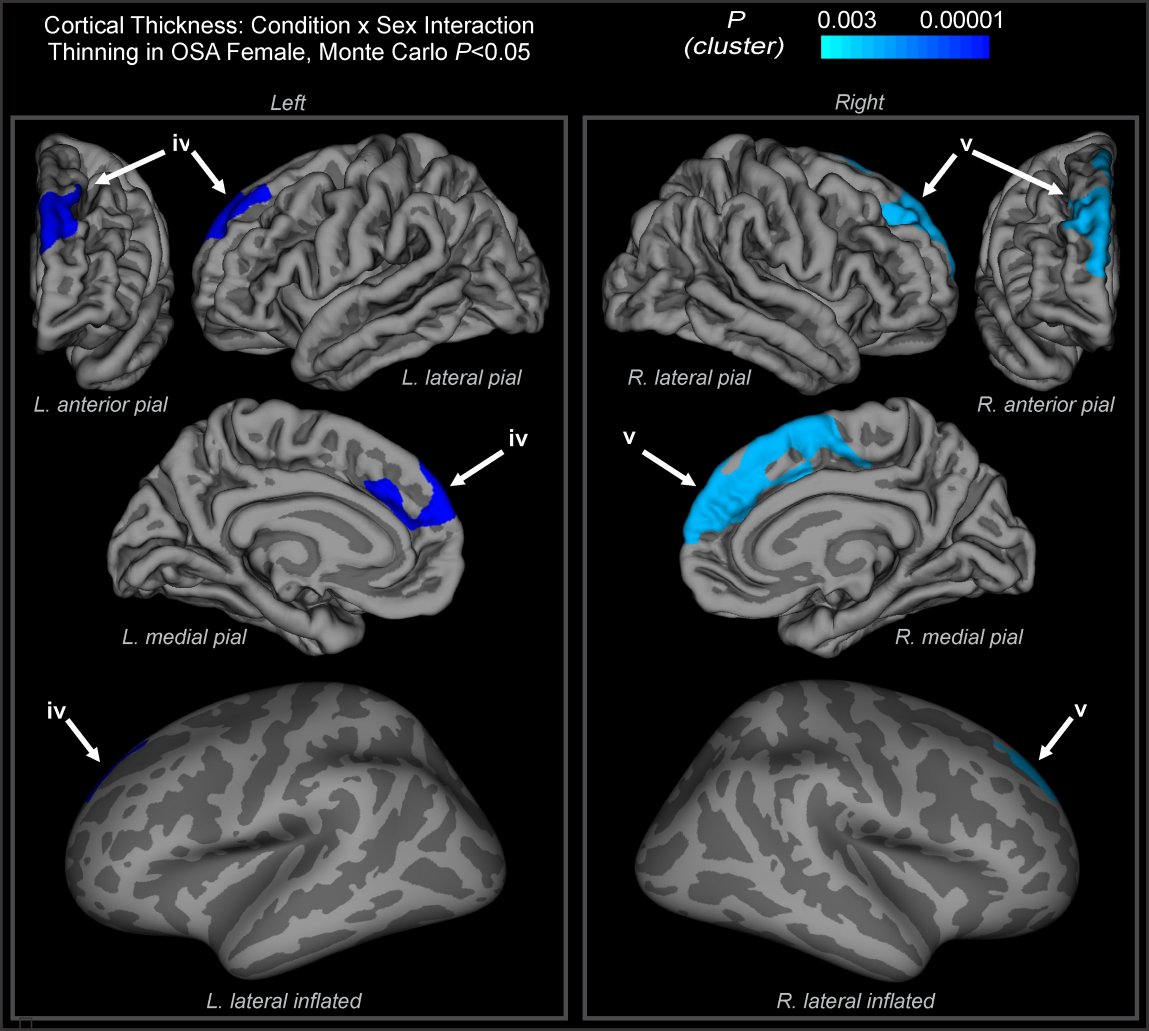
Sex-specific cortical thinning in OSA. Regions showing an interaction of condition and sex, with blue areas showing cortical thinning in OSA females (*p* < 0.05, Monte Carlo correction for multiple comparisons). Color scale shows the cluster-level *p* value. Pial and inflated views depicted, with light gray indicating gyri and dark gray sulci. (iv) left hemisphere cluster extending into superior medial frontal cortex, superior aspect of mid-to-anterior cingulate; (v) right hemisphere cluster extending into superior medial frontal cortex, prefrontal cortex, superior aspect of mid-to-anterior cingulate, supplementary motor cortex. See Table 3.

Significant cortical thinning appeared in the female OSA group compared to all other groups in two focal brain regions after the Monte Carlo cluster correction (*P* < 0.05). Thinning clusters were located bilaterally in the superior frontal lobe, in the left (Talairach peak: -8.5, 31.1, 28.3) and right hemisphere (Talairach peak: 6.6, 4.7, 57.3) (Table 3). Brain maps of the interaction effects between group and sex in mean thickness are shown for the left and right hemispheres (Fig 2). Quantitative data from each of these clusters are displayed in Table 3. No other interaction effects were present; that is, no areas showed male-specific cortical thinning in OSA (or lower thickness in control female or male groups).

**Table 3:** Interaction effect between condition and sex in mean thickness, as illustrated in Fig 2. Areas are labelled according to the Desikan[20] and Brodmann[70] atlases.

Anatomical Location of Cluster	Functional structures in cluster	Figure	Cluster size (mm^2^)	Peak (Talairach coordinates in mm)			
				X	Y	Z	P*
• L. superior frontal^a^ • (BA 9, 10) • L. Rostral middle frontal (BA 10)	• L. Superior frontal partial coverage executive decision making • L. Rostral middle frontal: apex of executive system underlying decision-making [74] • L. Cingulate (partial mid/partial anterior) adjacent (autonomic blood pressure regulation, cognitive decision-making/emotion)	Fig 2iv	1125	-8.5	31.1	28.3	0.04170
• R. Superior frontal^a^ • (BA 8, 9, 10) • R. rostral middle frontal (BA 10) • R. paracentral lobe (BA 6)	• R. Superior frontal-prefrontal cortex: extensive coverage of executive decision making area • R. Rostral middle frontal: prospective memory [75] • R. Cingulate (superior mid/partial anterior) adjacent (autonomic blood pressure regulation, cognitive decision-making) • R. Paracentral/precentral: supplementary motor area	Fig 2v	3088	6.6	4.7	57.3	0.00010

Abbreviations: L, left; R, right

a, FreeSurfer principal anatomical location of the peak (label of region most representative of area)

BA: Brodmann’s area

* cluster-wise P-value.

## Discussion

### Overview

Patients with OSA show cortical thinning in three regions, each covering multiple structures, with additional bilateral areas showing sex-specific effects in females. We found left hemisphere cortical thinning associated with OSA independent of sex in the precentral motor gyrus, the postcentral sensory gyrus, and temporal and insular cortices. Thinning in cortical regions in OSA differed by sex, with bilateral thinning appearing specifically in female OSA subjects in the left superior medial frontal region, extending into the rostral mid-frontal lobe, and in a similar area on the right superior frontal cortex, extending into both the rostral mid-frontal lobes and the anterior paracentral/precentral lobule. These differences from males may be related to sex-specific symptom patterns, especially cognitive symptoms that are more prominent in female OSA patients. The results add localized cortical atrophy to the brain characteristics that differ between males and females with OSA. Furthermore, an unanticipated finding was OSA thinning in regions associated with upper airway sensory and motor control. The thinning was especially noticeable in sensory and motor cortex, including upper airway, thoracic, abdominal and diaphragmatic regions.

### OSA effects: Relationships between affected areas and symptoms

A principal finding in OSA, independent of sex, was the cortical thinning in the motor and sensory areas of the upper airway musculature. Based on the motor homunculus, the damage within the left pre-central cortex in the OSA group represents regions that innervate the neck, face, lips, tongue, jaw muscles, as well as hands and arms [21]. Bilateral cortical damage was present within the somatosensory cortex in areas that innervate the face, upper and lower lip, tongue, pharynx, and nasal sensory areas [22]. In the left hemisphere, the region of damage extended into the superior parietal lobe, the supramarginal gyrus, the superior temporal gyrus, and the inferior parietal lobe. Progressive hypercapnia, hypoxia, and negative airway pressure enhance drive to upper airway dilator muscles [23–25]. Different muscle groups maintain upper airway patency, including muscles that control the tongue, palate, and hyoid [26]. The cortical thinning apparent in the sensory and motor integrative regions likely contributes to the atonia of genioglossal and upper airway pharyngeal dilator muscles in the presence of active diaphragmatic movements [26]. Being a cross-sectional study, we cannot distinguish whether the findings present cause or effect, and the cortical alterations may have preceded the development of OSA [27]. As mentioned above, one region showing indications of thinning regulates upper airway musculature, so if injury lead to atonia, the patterns seen her could contribute to the onset of the sleep disorder.

The cortical thinning of the insula may contribute to the sympathetic dysregulation in OSA. The insula helps to mediate sympathetic and parasympathetic outflow, and receives nociceptive and visceral sensory input to help integrate that outflow [28, 29]. The role of the insula in response to autonomic challenges has been described elsewhere in OSA and control populations [30–33]. The cortical thinning within the left insular cortex may contribute to the elevated sympathetic activity in OSA through reduction in inhibition of normal insular influences on the hypothalamus [34, 35].

### OSA by sex interactions: Relationships between affected areas and symptoms

The cortical damage in female OSA patients was localized bilaterally in the superior frontal regions, which have functions related to some sex-specific symptoms. Volume reductions in these areas could contribute to mood disturbances, such as depression [36]. Additionally, damage here may provide neuroanatomical substrates underlying disturbed executive function [37] and working memory [38]. The cortical thinning within the superior frontal regions extended into regions adjacent, or slightly overlapping the superior frontal gyrus, including the anterior cingulate cortex (ACC), which plays significant roles in executive processes of both cognition [39] and mood regulation [40], and projects to other limbic and prefrontal cortex sites [41]. However, depression-related areas are principally associated with the sub-genu of the ACC [42], while cortical thinning appeared only in the posterior aspect of the dorsal ACC; thus, the relationship of the enhanced depression signs in females to the thinning is not clear cut.

### OSA effects: Relationships with previous studies

The findings extend the existing body of OSA knowledge by demonstrating left motor and bilateral sensory strip cortical thinning in upper airway regions. The areas of cortical thinning also overlap with data from earlier studies showing gray matter impairments in OSA. Reduced regional gray matter volume occurs in the right post-central gyrus, a site involved in sensory regulation of the upper airway, and in the temporal lobe [43, 44]. Severe apneic patients have been previously shown to have showed localized cortical thinning in regions overlapping, or adjacent to the current findings in the insular cortex and inferior parietal lobe [45]. Reduced gray matter integrity also appears in the pre-central gyrus in severe OSA patients [46]. The variation between studies likely relates to several factors, including variation between samples in terms of OSA severity, co-morbidities, and sex distributions, as well as inherent heterogeneity of the condition of OSA.

OSA is associated with specific reductions in gray matter in some structures, while white matter exhibits less volume reduction or potentially volume increases. In addition to the present and earlier findings of specific thinning in the cortex, additional studies, restricted to individual gray matter structures, showed volume reductions, including the mammillary bodies [47], sub-regions of the putamen [48], and the hippocampus in a small sample [49]. White matter volume has not been specifically assessed, but changes could arise from astrocyte activation to hypoxia and increased water content [50]. For brain imaging studies, such processes would confound the standard voxel-based morphometry (VBM) analyses of regional gray matter volume, because VBM calculations are performed on a whole-brain scan, and influences of white matter structure will be present in determination of gray matter volume of the analyzed image. This confound could underlie the weaker findings of the VBM studies in the literature.

Sex is a factor that influences findings of OSA-related brain changes. Our group’s work suggests OSA has a greater impact in females [4], so it is possible male-only samples would have a lower magnitude of structural change. Indeed, a recent study of 27 male-only patients showed no OSA effects on regional gray matter volume differences, although that same study did demonstrate hypoxia-related volume reductions in temporal bilateral temporal regions [51], An earlier study of 27 OSA males also showed no large effect of OSA on gray matter volume [52], although when combined with a larger sample, volume reductions were apparent [43].

### OSA by sex interactions: Relationship with previous studies

The majority of studies of brain structure in OSA to date have either been only in males [44–46, 51, 52], or have included sex as a nuisance variable, but did not evaluate findings in males and females separately [47, 53]. Our earlier study of white matter integrity did show sex-specific differences [4], with greater reduction in axonal integrity in female OSA than male OSA patients. Using a diffusion tensor imaging (DTI)-derived measure to assess axonal structural integrity, namely fractional anisotropy (FA), OSA patients show extensive structural white matter changes. Affected axonal bundles include interconnecting fibers to limbic structures, such as the bilateral cingulum and right stria terminalis, and axons to bilateral frontal and parietal cortices and the left superior cerebellar peduncle [54]. The cortical damage associated with OSA to the left superior parietal cortex, the left inferior parietal cortex, and the bilateral pre- and post-central gyri may either be a cause of, or induced by, this white matter damage. However, only the superior frontal cortex showed sex-specific thinning associated with female OSA.

### Underlying pathophysiology of cortical thinning

Cortical thickness correlates with the number of neurons in a given region of the cortex, and reduced thickness suggests the presence of neuronal damage [55]. Reduced cortical thickness can represent neuronal degeneration as well as loss of supporting glia. Such damage could arise from several processes, including hypoxia, impaired perfusion, inflammation, or inadequate nutrient support, such as insufficient thiamine and magnesium, essential for glucose metabolism; this mechanism of injury is suspect in other regions [47]. Intermittent hypoxia produces both molecular and cellular neuronal damage, with sex-specific effects [56–58], and likely directly contributes to some of the injury identified here [59]. Impaired perfusion and hemodynamic changes presumably also contribute to the cortical structural deficits in OSA patients through ischemic processes. Regional cerebral blood flow in OSA is reduced in major sensory and motor fiber systems and motor regulatory sites [60], and may lead to the cortical damage observed in the pre- and post-central gyri. Intermittent hypoxia inflammation-induced microglia result in neuron apoptosis and CNS dysfunction [61], and presumably, cortical thinning would appear at the end of such processes (since the early stages of inflammation would be expected to be associated with volume increases rather than decreases). Pre-clinical stages of common co-morbidities of OSA such as type-2 diabetes and hypertension may contribute to neural injury [52, 62], but whether such contributions to neural injury differ between sexes has not been examined.

## Limitations

A greater number of female OSA patients would have been preferable, but this was a secondary analysis as at the time of data collection sex-specific analyses were not planned. The OSA severity ranged from mild to very severe, with more mild patients in the female group, so the findings may have masked further sex differences, and variations with severity, especially given mild OSA (at least in males) may have limited clinical impact [63, 64]. While the cortical thickness-specific procedures are more sensitive to cortical changes than other techniques, the analysis is limited in spatial accuracy, as individual differences mean the necessary normalization (that is, matching individual brain scans to a reference template brain) will contain inaccuracies. A possible consequence of such limitations is that smaller regions impacted in OSA could have been missed. The possible inclusion of undiagnosed OSA in the control group may also have diluted the findings. The OSA subjects in the present study had, on average, fewer co-morbidities, lower medication usage, and lower BMI than the general OSA patient population, and therefore the findings may not generalize. The most likely difference in the general OSA population would be greater cortical thinning due to neurodegenerative consequences of comorbid conditions. Many symptoms with OSA and sex-specific associations such as age, BMI, and psychological symptoms are associated with changes to the brain [65–67], and a larger study could assess such effects in the OSA female and male populations; the group differences in these symptoms may have contributed to some of the effects seen. Handedness may have confounded the findings since both left and right-handed subjects were included. Since there were no differences in the proportions of right and left handed in OSA or control groups, the effect would likely be lower sensitivity to findings related to dominant handedness. The cerebellum was excluded from this analysis due to limitations of FreeSurfer software, but cerebellar cortex likely includes equivalent thinning given previous findings of reduced gray matter volume and axonal deficits in that region [43, 44, 46, 53, 68, 69]. With current sleep studies measures such as oxygen desaturation index we could assess the relationship between severity of intermittent hypoxia and cortical thinning; the AHI is known to be a poor indication of neurological symptoms [5].

### Conclusions

Newly-diagnosed OSA patients without major comorbidities showed cortical thinning in distinct regions of the cortex. One caveat is that polysomnography was not performed in all control subjects, so the results may be confounded by undiagnosed OSA in that group; such a confound would likely lead to higher false negatives. In particular, sensory and motor gyri in areas representing the upper airway and other respiratory control regions had thinner cortex in OSA relative to healthy control subjects. Distinct female and male patterns of OSA-related cortical thinning appeared, with thinning in female patients in the bilateral superior medial frontal and prefrontal cortices, which may underlie some of the sex differences in disease characteristics and comorbidities. In light of previously-shown gray and white matter changes in deeper brain structures of OSA patients, the CNS-related dysfunction in the condition likely arises from both poor axonal communication, due to the widespread white matter injury, and poor neural function, due to the localized gray matter injury. Damage to glia might also indirectly impair neural function. The alterations could arise from OSA directly, as well as from cormobidities and pathophysiology of the sleep disorder. These structural changes could include both long term neurodegeneration and short term processes, such as inflammation and glial activation. The latter should be reversible with treatment, but the degree to which brain function deficits are reversible is unclear.

## References

[1] Kilicarslan R, Alkan A, Sharifov R, Akkoyunlu ME, Aralasmak A, Kocer A, et al The effect of obesity on brain diffusion alteration in patients with obstructive sleep apnea. TheScientificWorldJournal. 2014;2014:768415 doi: 10.1155/2014/768415 ; PubMed Central PMCID: PMC3960565. 24729752 10.1155/2014/768415 PMC3960565

[2] Santarnecchi E, Sicilia I, Richiardi J, Vatti G, Polizzotto NR, Marino D, et al Altered cortical and subcortical local coherence in obstructive sleep apnea: a functional magnetic resonance imaging study. Journal of sleep research. 2013;22(3):337–47. doi: 10.1111/jsr.12006 . 23171248 10.1111/jsr.12006

[3] Somers VK, White DP, Amin R, Abraham WT, Costa F, Culebras A, et al Sleep apnea and cardiovascular disease: an American Heart Association/american College Of Cardiology Foundation Scientific Statement from the American Heart Association Council for High Blood Pressure Research Professional Education Committee, Council on Clinical Cardiology, Stroke Council, and Council On Cardiovascular Nursing. In collaboration with the National Heart, Lung, and Blood Institute National Center on Sleep Disorders Research (National Institutes of Health). Circulation. 2008;118(10):1080–111. doi: 10.1161/CIRCULATIONAHA.107.189375 . 18725495 10.1161/CIRCULATIONAHA.107.189375

[4] Macey PM, Kumar R, Yan-Go FL, Woo MA, Harper RM. Sex differences in white matter alterations accompanying obstructive sleep apnea. Sleep. 2012;35(12):1603–13. doi: 10.5665/sleep.2228 ; PubMed Central PMCID: PMC3490353. 23204603 10.5665/sleep.2228 PMC3490353

[5] Macey PM, Woo MA, Kumar R, Cross RL, Harper RM. Relationship between obstructive sleep apnea severity and sleep, depression and anxiety symptoms in newly-diagnosed patients. PLoS One. 2010;5(4):e10211 Epub 2010/04/27. doi: 10.1371/journal.pone.0010211 ; PubMed Central PMCID: PMCPMC2855711. 20419135 10.1371/journal.pone.0010211 PMC2855711

[6] Young T, Evans L, Finn L, Palta M. Estimation of the clinically diagnosed proportion of sleep apnea syndrome in middle-aged men and women. Sleep. 1997;20(9):705–6. . 9406321 10.1093/sleep/20.9.705

[7] Shepertycky MR, Banno K, Kryger MH. Differences between men and women in the clinical presentation of patients diagnosed with obstructive sleep apnea syndrome. Sleep. 2005;28(3):309–14. Epub 2005/09/22. . 16173651

[8] McCall WV, Harding D, O'Donovan C. Correlates of depressive symptoms in patients with obstructive sleep apnea. Journal of clinical sleep medicine: JCSM: official publication of the American Academy of Sleep Medicine. 2006;2(4):424–6. . 17557471

[9] Asghari A, Mohammadi F, Kamrava SK, Tavakoli S, Farhadi M. Severity of depression and anxiety in obstructive sleep apnea syndrome. Eur Arch Otorhinolaryngol. 2012;269(12):2549–53. doi: 10.1007/s00405-012-1942-6 . 22298252 10.1007/s00405-012-1942-6

[10] Young T, Hutton R, Finn L, Badr S, Palta M. The gender bias in sleep apnea diagnosis. Are women missed because they have different symptoms? Archives of internal medicine. 1996;156(21):2445–51. Epub 1996/11/25. . 8944737

[11] Simons M, Misgeld T, Kerschensteiner M. A unified cell biological perspective on axon-myelin injury. The Journal of cell biology. 2014;206(3):335–45. doi: 10.1083/jcb.201404154 ; PubMed Central PMCID: PMC4121977. 25092654 10.1083/jcb.201404154 PMC4121977

[12] Fischl B. FreeSurfer. NeuroImage. 2012;62(2):774–81. doi: 10.1016/j.neuroimage.2012.01.021 ; PubMed Central PMCID: PMC3685476. 22248573 10.1016/j.neuroimage.2012.01.021 PMC3685476

[13] Kim JS, Youn J, Yang JJ, Lee DK, Lee JM, Kim ST, et al Topographic distribution of cortical thinning in subtypes of multiple system atrophy. Parkinsonism & related disorders. 2013;19(11):970–4. doi: 10.1016/j.parkreldis.2013.06.012 . 23867866 10.1016/j.parkreldis.2013.06.012

[14] Webster JB, Bell KR, Hussey JD, Natale TK, Lakshminarayan S. Sleep apnea in adults with traumatic brain injury: a preliminary investigation. Archives of physical medicine and rehabilitation. 2001;82(3):316–21. doi: 10.1053/apmr.2001.20840 . 11245752 10.1053/apmr.2001.20840

[15] Sleep-related breathing disorders in adults: recommendations for syndrome definition and measurement techniques in clinical research. The Report of an American Academy of Sleep Medicine Task Force. Sleep. 1999;22(5):667–89. . 10450601

[16] Buysse DJ, Reynolds CF 3rd, Monk TH, Berman SR, Kupfer DJ. The Pittsburgh Sleep Quality Index: a new instrument for psychiatric practice and research. Psychiatry Res. 1989;28(2):193–213. Epub 1989/05/01. doi: 0165-1781(89)90047-4 [pii]. . 2748771 10.1016/0165-1781(89)90047-4

[17] Beck A, Steer R, Brown G. Manual for the Beck Depression Inventory-II San Antonio, Texas: The Psychological Corporation; 1996.

[18] Beck AT, Epstein N, Brown G, Steer RA. An inventory for measuring clinical anxiety: psychometric properties. J Consult Clin Psychol. 1988;56(6):893–7. Epub 1988/12/01. . 3204199 10.1037//0022-006x.56.6.893

[19] Dale AM, Fischl B, Sereno MI. Cortical surface-based analysis. I. Segmentation and surface reconstruction. NeuroImage. 1999;9(2):179–94. Epub 1999/02/05. doi: S1053-8119(98)90395-0 [pii] doi: 10.1006/nimg.1998.0395 . 9931268 10.1006/nimg.1998.0395

[20] Desikan RS, Segonne F, Fischl B, Quinn BT, Dickerson BC, Blacker D, et al An automated labeling system for subdividing the human cerebral cortex on MRI scans into gyral based regions of interest. NeuroImage. 2006;31(3):968–80. doi: 10.1016/j.neuroimage.2006.01.021 . 16530430 10.1016/j.neuroimage.2006.01.021

[21] Metman LV, Bellevich JS, Jones SM, Barber MD, Streletz LJ. Topographic mapping of human motor cortex with transcranial magnetic stimulation: Homunculus revisited. Brain topography. 1993;6(1):13–9. . 8260321 10.1007/BF01234122

[22] Parpia P. Reappraisal of the somatosensory homunculus and its discontinuities. Neural computation. 2011;23(12):3001–15. doi: 10.1162/NECO_a_00179 . 21732862 10.1162/NECO_a_00179

[23] Brouillette RT, Thach BT. Control of genioglossus muscle inspiratory activity. Journal of applied physiology: respiratory, environmental and exercise physiology. 1980;49(5):801–8. doi: 10.1152/jappl.1980.49.5.801 . 6776078 10.1152/jappl.1980.49.5.801

[24] Onal E, Lopata M, O'Connor TD. Diaphragmatic and genioglossal electromyogram responses to CO2 rebreathing in humans. Journal of applied physiology: respiratory, environmental and exercise physiology. 1981;50(5):1052–5. doi: 10.1152/jappl.1981.50.5.1052 . 6785263 10.1152/jappl.1981.50.5.1052

[25] Horner RL, Innes JA, Holden HB, Guz A. Afferent pathway(s) for pharyngeal dilator reflex to negative pressure in man: a study using upper airway anaesthesia. The Journal of physiology. 1991;436:31–44. ; PubMed Central PMCID: PMC1181492. 2061834 10.1113/jphysiol.1991.sp018537 PMC1181492

[26] Deegan PC, McNicholas WT. Pathophysiology of obstructive sleep apnoea. The European respiratory journal. 1995;8(7):1161–78. . 7589402 10.1183/09031936.95.08071161

[27] Gozal D. The brain in sleep-disordered breathing: is it the chicken or is it the egg? Am J Respir Crit Care Med. 2002;166(10):1305–6. doi: 10.1164/rccm.2208005 . 12421736 10.1164/rccm.2208005

[28] Mazzola L, Faillenot I, Barral FG, Mauguiere F, Peyron R. Spatial segregation of somato-sensory and pain activations in the human operculo-insular cortex. NeuroImage. 2012;60(1):409–18. doi: 10.1016/j.neuroimage.2011.12.072 . 22245639 10.1016/j.neuroimage.2011.12.072

[29] Cechetto DF, Chen SJ. Subcortical sites mediating sympathetic responses from insular cortex in rats. The American journal of physiology. 1990;258(1 Pt 2):R245–55. doi: 10.1152/ajpregu.1990.258.1.R245 . 2301638 10.1152/ajpregu.1990.258.1.R245

[30] Harper RM, Macey PM, Henderson LA, Woo MA, Macey KE, Frysinger RC, et al fMRI responses to cold pressor challenges in control and obstructive sleep apnea subjects. J Appl Physiol. 2003;94(4):1583–95. Epub 2003/01/07. doi: 10.1152/japplphysiol.00881.2002 [pii]. . 12514164 10.1152/japplphysiol.00881.2002

[31] Henderson LA, Woo MA, Macey PM, Macey KE, Frysinger RC, Alger JR, et al Neural responses during Valsalva maneuvers in obstructive sleep apnea syndrome. J Appl Physiol. 2003;94(3):1063–74. doi: 10.1152/japplphysiol.00702.2002 . 12433858 10.1152/japplphysiol.00702.2002

[32] Macey KE, Macey PM, Woo MA, Henderson LA, Frysinger RC, Harper RK, et al Inspiratory loading elicits aberrant fMRI signal changes in obstructive sleep apnea. Respir Physiol Neurobiol. 2006;151(1):44–60. Epub 2005/07/05. doi: S1569-9048(05)00154-0 [pii] doi: 10.1016/j.resp.2005.05.024 . 15993658 10.1016/j.resp.2005.05.024

[33] Macey PM, Wu P, Kumar R, Ogren JA, Richardson HL, Woo MA, et al Differential responses of the insular cortex gyri to autonomic challenges. Auton Neurosci. 2012;168(1–2):72–81. Epub 2012/02/22. doi: 10.1016/j.autneu.2012.01.009 . 22342370 10.1016/j.autneu.2012.01.009 PMC4077282

[34] Tsumori T, Yokota S, Qin Y, Oka T, Yasui Y. A light and electron microscopic analysis of the convergent insular cortical and amygdaloid projections to the posterior lateral hypothalamus in the rat, with special reference to cardiovascular function. Neuroscience research. 2006;56(3):261–9. Epub 2006/08/29. doi: 10.1016/j.neures.2006.07.005 . 16935375 10.1016/j.neures.2006.07.005

[35] Saper CB. Convergence of autonomic and limbic connections in the insular cortex of the rat. J Comp Neurol. 1982;210(2):163–73. doi: 10.1002/cne.902100207 . 7130477 10.1002/cne.902100207

[36] Joseph R. Frontal lobe psychopathology: mania, depression, confabulation, catatonia, perseveration, obsessive compulsions, and schizophrenia. Psychiatry. 1999;62(2):138–72. . 10420428 10.1080/00332747.1999.11024862

[37] Ball G, Stokes PR, Rhodes RA, Bose SK, Rezek I, Wink AM, et al Executive functions and prefrontal cortex: a matter of persistence? Frontiers in systems neuroscience. 2011;5:3 doi: 10.3389/fnsys.2011.00003 ; PubMed Central PMCID: PMC3031025. 21286223 10.3389/fnsys.2011.00003 PMC3031025

[38] du Boisgueheneuc F, Levy R, Volle E, Seassau M, Duffau H, Kinkingnehun S, et al Functions of the left superior frontal gyrus in humans: a lesion study. Brain: a journal of neurology. 2006;129(Pt 12):3315–28. doi: 10.1093/brain/awl244 . 16984899 10.1093/brain/awl244

[39] Carter CS, Macdonald AM, Botvinick M, Ross LL, Stenger VA, Noll D, et al Parsing executive processes: strategic vs. evaluative functions of the anterior cingulate cortex. Proceedings of the National Academy of Sciences of the United States of America. 2000;97(4):1944–8. ; PubMed Central PMCID: PMC26541. 10677559 10.1073/pnas.97.4.1944 PMC26541

[40] Drevets WC, Savitz J, Trimble M. The subgenual anterior cingulate cortex in mood disorders. CNS spectrums. 2008;13(8):663–81. ; PubMed Central PMCID: PMC2729429. 18704022 10.1017/s1092852900013754 PMC2729429

[41] Stevens FL, Hurley RA, Taber KH. Anterior cingulate cortex: unique role in cognition and emotion. The Journal of neuropsychiatry and clinical neurosciences. 2011;23(2):121–5. doi: 10.1176/appi.neuropsych.23.2.121 . 21677237 10.1176/jnp.23.2.jnp121

[42] Drevets WC, Ongur D, Price JL. Neuroimaging abnormalities in the subgenual prefrontal cortex: implications for the pathophysiology of familial mood disorders. Molecular psychiatry. 1998;3(3):220–6, 190–1. Epub 1998/07/22. . 9672897 10.1038/sj.mp.4000370

[43] Morrell MJ, Jackson ML, Twigg GL, Ghiassi R, McRobbie DW, Quest RA, et al Changes in brain morphology in patients with obstructive sleep apnoea. Thorax. 2010;65(10):908–14. Epub 2010/09/24. doi: 10.1136/thx.2009.126730 . 20861295 10.1136/thx.2009.126730

[44] Macey PM, Henderson LA, Macey KE, Alger JR, Frysinger RC, Woo MA, et al Brain morphology associated with obstructive sleep apnea. Am J Respir Crit Care Med. 2002;166(10):1382–7. doi: 10.1164/rccm.200201-050OC . 12421746 10.1164/rccm.200201-050OC

[45] Joo EY, Jeon S, Kim ST, Lee JM, Hong SB. Localized cortical thinning in patients with obstructive sleep apnea syndrome. Sleep. 2013;36(8):1153–62. doi: 10.5665/sleep.2876 ; PubMed Central PMCID: PMC3700712. 23904675 10.5665/sleep.2876 PMC3700712

[46] Joo EY, Tae WS, Lee MJ, Kang JW, Park HS, Lee JY, et al Reduced brain gray matter concentration in patients with obstructive sleep apnea syndrome. Sleep. 2010;33(2):235–41. Epub 2010/02/24. ; PubMed Central PMCID: PMC2817910. 20175407 10.1093/sleep/33.2.235 PMC2817910

[47] Kumar R, Birrer BV, Macey PM, Woo MA, Gupta RK, Yan-Go FL, et al Reduced mammillary body volume in patients with obstructive sleep apnea. Neurosci Lett. 2008;438(3):330–4. Epub 2008/05/20. doi: S0304-3940(08)00573-9 [pii] doi: 10.1016/j.neulet.2008.04.071 . 18486338 10.1016/j.neulet.2008.04.071

[48] Kumar R, Farahvar S, Ogren JA, Macey PM, Thompson PM, Woo MA, et al Brain putamen volume changes in newly-diagnosed patients with obstructive sleep apnea. NeuroImage Clinical. 2014;4:383–91. doi: 10.1016/j.nicl.2014.01.009 ; PubMed Central PMCID: PMC3930100. 24567910 10.1016/j.nicl.2014.01.009 PMC3930100

[49] Morrell MJ, McRobbie DW, Quest RA, Cummin AR, Ghiassi R, Corfield DR. Changes in brain morphology associated with obstructive sleep apnea. Sleep Med. 2003;4(5):451–4. . 14592287 10.1016/s1389-9457(03)00159-x

[50] Baronio D, Martinez D, Fiori CZ, Bambini-Junior V, Forgiarini LF, Pase da Rosa D, et al Altered aquaporins in the brains of mice submitted to intermittent hypoxia model of sleep apnea. Respir Physiol Neurobiol. 2013;185(2):217–21. doi: 10.1016/j.resp.2012.10.012 . 23123204 10.1016/j.resp.2012.10.012

[51] Huynh NT, Prilipko O, Kushida CA, Guilleminault C. Volumetric Brain Morphometry Changes in Patients with Obstructive Sleep Apnea Syndrome: Effects of CPAP Treatment and Literature Review. Frontiers in neurology. 2014;5:58 doi: 10.3389/fneur.2014.00058 ; PubMed Central PMCID: PMC4010762. 24808886 10.3389/fneur.2014.00058 PMC4010762

[52] O'Donoghue FJ, Briellmann RS, Rochford PD, Abbott DF, Pell GS, Chan CH, et al Cerebral structural changes in severe obstructive sleep apnea. Am J Respir Crit Care Med. 2005;171(10):1185–90. doi: 10.1164/rccm.200406-738OC . 15699018 10.1164/rccm.200406-738OC

[53] Macey PM, Kumar R, Woo MA, Valladares EM, Yan-Go FL, Harper RM. Brain structural changes in obstructive sleep apnea. Sleep. 2008;31(7):967–77. Epub 2008/07/26. ; PubMed Central PMCID: PMCPMC2491498. 18652092 PMC2491498

[54] Macey PM. Is brain injury in obstructive sleep apnea reversible? Sleep. 2012;35(1):9–10. Epub 2012/01/05. doi: 10.5665/sleep.1572 ; PubMed Central PMCID: PMC3242693. 22215912 10.5665/sleep.1572 PMC3242693

[55] Shefer VF. Absolute number of neurons and thickness of the cerebral cortex during aging, senile and vascular dementia, and Pick's and Alzheimer's diseases. Neuroscience and behavioral physiology. 1973;6(4):319–24. . 4781784 10.1007/BF01182672

[56] Sanfilippo-Cohn B, Lai S, Zhan G, Fenik P, Pratico D, Mazza E, et al Sex differences in susceptibility to oxidative injury and sleepiness from intermittent hypoxia. Sleep. 2006;29(2):152–9. Epub 2006/02/24. . 16494082 10.1093/sleep/29.2.152

[57] Nijboer CH, Kavelaars A, van Bel F, Heijnen CJ, Groenendaal F. Gender-dependent pathways of hypoxia-ischemia-induced cell death and neuroprotection in the immature P3 rat. Developmental neuroscience. 2007;29(4–5):385–92. doi: 10.1159/000105479 . 17762206 10.1159/000105479

[58] Nijboer CH, Groenendaal F, Kavelaars A, Hagberg HH, van Bel F, Heijnen CJ. Gender-specific neuroprotection by 2-iminobiotin after hypoxia-ischemia in the neonatal rat via a nitric oxide independent pathway. J Cereb Blood Flow Metab. 2007;27(2):282–92. Epub 2006/06/01. doi: 9600342 [pii] doi: 10.1038/sj.jcbfm.9600342 . 16736041 10.1038/sj.jcbfm.9600342

[59] Yang Q, Wang Y, Feng J, Cao J, Chen B. Intermittent hypoxia from obstructive sleep apnea may cause neuronal impairment and dysfunction in central nervous system: the potential roles played by microglia. Neuropsychiatric disease and treatment. 2013;9:1077–86. doi: 10.2147/NDT.S49868 ; PubMed Central PMCID: PMC3742344. 23950649 10.2147/NDT.S49868 PMC3742344

[60] Yadav SK, Kumar R, Macey PM, Richardson HL, Wang DJ, Woo MA, et al Regional cerebral blood flow alterations in obstructive sleep apnea. Neurosci Lett. 2013;555:159–64. doi: 10.1016/j.neulet.2013.09.033 ; PubMed Central PMCID: PMC3891908. 24076138 10.1016/j.neulet.2013.09.033 PMC3891908

[61] Block ML, Hong JS. Microglia and inflammation-mediated neurodegeneration: multiple triggers with a common mechanism. Progress in neurobiology. 2005;76(2):77–98. doi: 10.1016/j.pneurobio.2005.06.004 . 16081203 10.1016/j.pneurobio.2005.06.004

[62] Harper RM, Macey PM, Kumar R, Woo MA. Neural Injury in Diabetic Versus Non-Diabetic Obstructive Sleep Apnea Patients: A Pilot Study. Sleep. 2009;32:A341–A. PubMed PMID: ISI:000265542001395.

[63] Littner MR. Mild obstructive sleep apnea syndrome should not be treated. Con. J Clin Sleep Med. 2007;3(3):263–4. Epub 2007/06/15. ; PubMed Central PMCID: PMC2564770. 17561592 PMC2564770

[64] Anttalainen U, Polo O, Saaresranta T. Is 'MILD' sleep-disordered breathing in women really mild? Acta Obstet Gynecol Scand. 2010;89(5):605–11. Epub 2010/04/29. doi: 10.3109/00016341003681249 . 20423273 10.3109/00016341003681249

[65] Pink A, Przybelski SA, Krell-Roesch J, Stokin GB, Roberts RO, Mielke MM, et al Cortical Thickness and Depressive Symptoms in Cognitively Normal Individuals: The Mayo Clinic Study of Aging. J Alzheimers Dis. 2017 doi: 10.3233/JAD-170041 . 28550256 10.3233/JAD-170041 PMC5713905

[66] Gennatas ED, Avants BB, Wolf DH, Satterthwaite TD, Ruparel K, Ciric R, et al Age-Related Effects and Sex Differences in Gray Matter Density, Volume, Mass, and Cortical Thickness from Childhood to Young Adulthood. J Neurosci. 2017;37(20):5065–73. doi: 10.1523/JNEUROSCI.3550-16.2017 ; PubMed Central PMCID: PMCPMC5444192. 28432144 10.1523/JNEUROSCI.3550-16.2017 PMC5444192

[67] Marques-Iturria I, Pueyo R, Garolera M, Segura B, Junque C, Garcia-Garcia I, et al Frontal cortical thinning and subcortical volume reductions in early adulthood obesity. Psychiatry Res. 2013;214(2):109–15. doi: 10.1016/j.pscychresns.2013.06.004 . 24041490 10.1016/j.pscychresns.2013.06.004

[68] Kumar R, Pham TT, Macey PM, Woo MA, Yan-Go FL, Harper RM. Abnormal myelin and axonal integrity in recently diagnosed patients with obstructive sleep apnea. Sleep. 2014;37(4):723–32. doi: 10.5665/sleep.3578 ; PubMed Central PMCID: PMCPMC4044745. 24899761 10.5665/sleep.3578 PMC4044745

[69] Kumar R, Chavez AS, Macey PM, Woo MA, Yan-Go FL, Harper RM. Altered global and regional brain mean diffusivity in patients with obstructive sleep apnea. J Neurosci Res. 2012;90(10):2043–52. doi: 10.1002/jnr.23083 ; PubMed Central PMCID: PMC3418429. 22715089 10.1002/jnr.23083 PMC3418429

[70] Brodmann K. Vergleichende Lokalisationslehre der Grosshirnrinde in ihren Prinzipien dargestellt auf Grund des Zellenbaues Leipzig: Barth; 1909 x, 324 p. p.

[71] Singh-Curry V, Husain M. The functional role of the inferior parietal lobe in the dorsal and ventral stream dichotomy. Neuropsychologia. 2009;47(6):1434–48. doi: 10.1016/j.neuropsychologia.2008.11.033 ; PubMed Central PMCID: PMC2697316. 19138694 10.1016/j.neuropsychologia.2008.11.033 PMC2697316

[72] Radua J, Phillips ML, Russell T, Lawrence N, Marshall N, Kalidindi S, et al Neural response to specific components of fearful faces in healthy and schizophrenic adults. NeuroImage. 2010;49(1):939–46. doi: 10.1016/j.neuroimage.2009.08.030 . 19699306 10.1016/j.neuroimage.2009.08.030

[73] Bonner MF, Price AR. Where is the anterior temporal lobe and what does it do? The Journal of neuroscience: the official journal of the Society for Neuroscience. 2013;33(10):4213–5. doi: 10.1523/JNEUROSCI.0041-13.2013 ; PubMed Central PMCID: PMC3632379. 23467339 10.1523/JNEUROSCI.0041-13.2013 PMC3632379

[74] Koechlin E, Hyafil A. Anterior prefrontal function and the limits of human decision-making. Science. 2007;318(5850):594–8. doi: 10.1126/science.1142995 . 17962551 10.1126/science.1142995

[75] Burgess PW, Scott SK, Frith CD. The role of the rostral frontal cortex (area 10) in prospective memory: a lateral versus medial dissociation. Neuropsychologia. 2003;41(8):906–18. . 12667527 10.1016/s0028-3932(02)00327-5

